# (*E*)-2,3-Bis[(*E*)-benzyl­idene­amino]­but-2-enedinitrile

**DOI:** 10.1107/S1600536812005363

**Published:** 2012-02-10

**Authors:** Matthew P. Akerman, Sarah J. Maher

**Affiliations:** a School of Chemistry and Physics, University of KwaZulu-Natal, Private Bag X01, Scottsville, Pietermaritzburg 3209, South Africa

## Abstract

The asymmetric unit of the title compound, C_18_H_12_N_4_, consists of a half-mol­ecule, where the two halves of the mol­ecule are related by inversion symmetry. The mol­ecule is effectively planar, with the largest deviation from the 22-atom mean plane, measuring 0.024 (2) Å, exhibited by the *ortho*-C atom of the phenyl ring. The crystal structure exhibits π-stacking, with an inter­planar spacing of 3.431 (3) Å.

## Related literature

For applications of the title mol­ecule as a semiconductor, see: Tanaka *et al.* (2009 ▶). For applications of the title compound and its various derivatives as a dye, see: Neumer (1977 ▶); Begland (1976 ▶). For the crystal structures of three di(azomethine) dyes with various substituents at the *para* position of the benzene ring of the title compound, see: Matsumoto *et al.* (2004 ▶). For a study of the nonlinear optics applications of both the title compound and the mono-condensation product, see: Das *et al.* (2001 ▶). For a review of the chemistry and reactions of the diamino­maleonitrile, see: Al-Azmi *et al.* (2003 ▶).
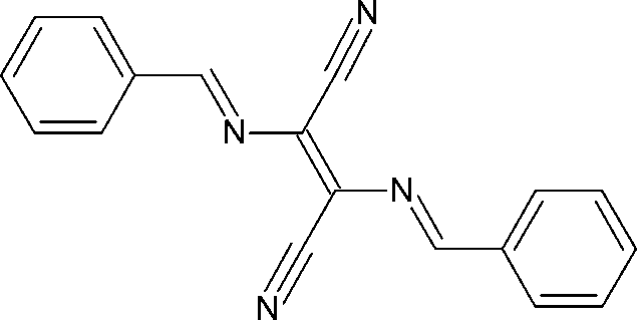


## Experimental

### 

#### Crystal data

C_18_H_12_N_4_
*M* *_r_* = 284.32
Triclinic, 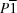

*a* = 6.389 (4) Å
*b* = 7.608 (5) Å
*c* = 8.311 (5) Å
α = 103.96 (5)°
β = 91.67 (5)°
γ = 102.97 (5)°
*V* = 380.6 (4) Å^3^
*Z* = 1
Mo *K*α radiation
μ = 0.08 mm^−1^
*T* = 295 K
0.50 × 0.20 × 0.10 mm

#### Data collection

Oxford Diffraction Xcalibur 2 CCD diffractometer
Absorption correction: multi-scan (Blessing, 1995 ▶) *T*_min_ = 0.963, *T*_max_ = 0.992
2824 measured reflections
1498 independent reflections
964 reflections with *I* > 2σ(*I*)
*R*_int_ = 0.025

#### Refinement

*R*[*F* ^2^ > 2σ(*F* ^2^)] = 0.045
*wR*(*F* ^2^) = 0.117
*S* = 0.89
1498 reflections
100 parameters
H-atom parameters constrained
Δρ_max_ = 0.11 e Å^−3^
Δρ_min_ = −0.21 e Å^−3^

### 

Data collection: *CrysAlis CCD* (Oxford Diffraction, 2008 ▶); cell refinement: *CrysAlis CCD*; data reduction: *CrysAlis RED* (Oxford Diffraction, 2008 ▶); program(s) used to solve structure: *SHELXS97* (Sheldrick, 2008 ▶); program(s) used to refine structure: *SHELXL97* (Sheldrick, 2008 ▶); molecular graphics: *ORTEP-3* (Farrugia, 1997 ▶); software used to prepare material for publication: *publCIF* (Westrip, 2010 ▶).

## Supplementary Material

Crystal structure: contains datablock(s) I, global. DOI: 10.1107/S1600536812005363/ez2280sup1.cif

Structure factors: contains datablock(s) I. DOI: 10.1107/S1600536812005363/ez2280Isup2.hkl

Supplementary material file. DOI: 10.1107/S1600536812005363/ez2280Isup3.mol

Supplementary material file. DOI: 10.1107/S1600536812005363/ez2280Isup4.cml

Additional supplementary materials:  crystallographic information; 3D view; checkCIF report

## Figures and Tables

**Fig. 1.**
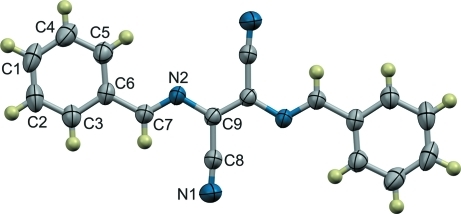
Labelled thermal ellipsoid plot of (1) (50% probability surfaces). Hydrogen atoms have been rendered as spheres of arbitrary radius.

## supplementary crystallographic information

### Comment

Di(azomethine) compounds derived from diaminomaleonitrile (DAMN) have been extensively studied for their application as dyes, semiconductors and in non-linear optics (Tanaka *et al.*, 2009; Neumer, 1977; Begland, 1976; Matsumoto *et al.*, 2004 and Das *et al.*, 2001). The title compound is interesting in that although it is orientated in a *trans* configuration about the ethylene group of the di(azomethine) linkage the DAMN synthon is exclusively *cis* about this bond. It is proposed that at elevated temperatures during the chemical synthesis an equilibrium is established between the *cis* and *trans* isomers of DAMN in solution. Although it has been shown that the *cis* isomer of DAMN is lower in energy than the *trans* isomer (Al-Azmi *et al.*, 2003) it is likely that non-bonded repulsion between the hydrogen atoms in the *ortho* positions of (1) would result in the *trans* isomer of (1) having a lower energy than the *cis* isomer. The double condensation reaction required to form (1) is therefore more likely to take place with the *trans* isomer of DAMN in solution; the *trans* isomer of (1) would thus be the major product.

Compound (1) crystallized in the triclinic space group P1 with a half molecule in the asymmetric unit. The two halves of the molecule are related by inversion symmetry with an inversion centre at the midpoint of the C═C double bond of the di(azomethine) linkage unit. The molecule is effectively planar with the largest deviations from the 22-atom mean plane exhibited by the *ortho* C-atoms C3 and C5, measuring 0.022 (2) and 0.024 (2) Å, respectively. The structure shows that adjacent molecules are parallel, but are in a staggered configuration. There appear to be π-π interactions between adjacent molecules with an interplanar spacing of 3.431 (3) Å. The structure shows no genuine hydrogen bonding as all interactions are longer than the sum of their van der Waals radii. The lack of meaningful hydrogen bonds is likely due to a lack of good H-bond donors, as both the cyanide groups and the imine nitrogen atoms are potentially good H-bond acceptors.

### Experimental

Benzaldehyde (0.500 g, 4.7 mmol), *cis*-diaminomaleonitrile (0.255 g, 2.4 mmol) and a catalytic amount of piperidine were dissolved in dry toluene (100 ml) and the solution brought to reflux for four hours. Water was continuously removed from the reaction *via* a Dean and Stark apparatus. The solvent was removed by rotary evaporation under reduced pressure and the resulting solid dissolved in a minimum of dichloromethane. The desired product was obtained by column chromatography on silica gel, using dichloromethane as the eluent. Single crystals were grown by slow evaporation of the eluent. Yield (0.413 g, 62%). ^1Ĥ NMR (500 MHz, CD3CN): 7.52 (t, 4H, m-phenyl), 7.60 (t, 2H, p-phenyl), 8.02 (d, 4H, o-phenyl), 8.75 (s, 2H, imine).

### Refinement

The positions of all hydrogen atoms were calculated using the standard riding model of *SHELXL97* with C—H(aromatic) distances of 0.93 Å and *U*_iso_ = 1.2 *U*_eq_.

### Crystal data

**Table:**

C_18_H_12_N_4_	*Z* = 1
*M**_r_* = 284.32	*F*(000) = 148
Triclinic, *P*1	*D*_x_ = 1.241 Mg m^−^^3^
Hall symbol: -P 1	Mo *K*α radiation, λ = 0.71073 Å
*a* = 6.389 (4) Å	Cell parameters from 2824 reflections
*b* = 7.608 (5) Å	θ = 3.3–26°
*c* = 8.311 (5) Å	µ = 0.08 mm^−^^1^
α = 103.96 (5)°	*T* = 295 K
β = 91.67 (5)°	Needle, yellow
γ = 102.97 (5)°	0.50 × 0.20 × 0.10 mm
*V* = 380.6 (4) Å^3^	

### Data collection

**Table:**

Oxford Diffraction Xcalibur 2 CCD diffractometer	1498 independent reflections
Radiation source: fine-focus sealed tube	964 reflections with *I* > 2σ(*I*)
Graphite monochromator	*R*_int_ = 0.025
ω scans at fixed θ angles	θ_max_ = 26.1°, θ_min_ = 3.3°
Absorption correction: multi-scan (Blessing, 1995)	*h* = −6→7
*T*_min_ = 0.963, *T*_max_ = 0.992	*k* = −9→9
2824 measured reflections	*l* = −9→10

### Refinement

**Table:**

Refinement on *F*^2^	Primary atom site location: structure-invariant direct methods
Least-squares matrix: full	Secondary atom site location: difference Fourier map
*R*[*F*^2^ > 2σ(*F*^2^)] = 0.045	Hydrogen site location: inferred from neighbouring sites
*wR*(*F*^2^) = 0.117	H-atom parameters constrained
*S* = 0.89	*w* = 1/[σ^2^(*F*_o_^2^) + (0.0717*P*)^2^] where *P* = (*F*_o_^2^ + 2*F*_c_^2^)/3
1498 reflections	(Δ/σ)_max_ < 0.001
100 parameters	Δρ_max_ = 0.11 e Å^−^^3^
0 restraints	Δρ_min_ = −0.21 e Å^−^^3^

### Special details

**Table:**

Experimental. ^1^H NMR (500 MHz, CD3CN): 7.52 (t, 4H, m-phenyl), 7.60 (t, 2H, p-phenyl), 8.02 (d, 4H, o-phenyl), 8.75 (s, 2H, imine).
Geometry. All s.u.'s (except the s.u. in the dihedral angle between two l.s. planes) are estimated using the full covariance matrix. The cell s.u.'s are taken into account individually in the estimation of s.u.'s in distances, angles and torsion angles; correlations between s.u.'s in cell parameters are only used when they are defined by crystal symmetry. An approximate (isotropic) treatment of cell s.u.'s is used for estimating s.u.'s involving l.s. planes.
Refinement. Refinement of *F*^2^ against ALL reflections. The weighted *R*-factor *wR* and goodness of fit *S* are based on *F*^2^, conventional *R*-factors *R* are based on *F*, with *F* set to zero for negative *F*^2^. The threshold expression of *F*^2^ > 2σ(*F*^2^) is used only for calculating *R*-factors(gt) *etc*. and is not relevant to the choice of reflections for refinement. *R*-factors based on *F*^2^ are statistically about twice as large as those based on *F*, and *R*- factors based on ALL data will be even larger.

### Fractional atomic coordinates and isotropic or equivalent isotropic displacement parameters (Å^2^)

**Table:**

	*x*	*y*	*z*	*U*_iso_*/*U*_eq_	
C1	−0.8068 (3)	0.8047 (3)	0.9287 (2)	0.0708 (5)	
H1	−0.9171	0.8241	0.9965	0.085*	
C2	−0.7329 (3)	0.9254 (3)	0.8351 (2)	0.0664 (5)	
H2	−0.7932	1.0265	0.8383	0.080*	
C3	−0.5686 (2)	0.8984 (2)	0.7353 (2)	0.0547 (4)	
H3	−0.5176	0.9818	0.6720	0.066*	
C4	−0.7201 (3)	0.6539 (3)	0.9241 (2)	0.0670 (5)	
H4	−0.7715	0.5720	0.9887	0.080*	
C5	−0.5573 (2)	0.6243 (2)	0.82398 (19)	0.0544 (4)	
H5	−0.4998	0.5215	0.8202	0.065*	
C6	−0.4787 (2)	0.74650 (19)	0.72903 (16)	0.0436 (4)	
C7	−0.3065 (2)	0.72118 (19)	0.62138 (17)	0.0451 (4)	
H7	−0.2608	0.8070	0.5592	0.054*	
C8	0.0179 (2)	0.7027 (2)	0.40742 (18)	0.0469 (4)	
C9	−0.0504 (2)	0.57010 (17)	0.50538 (16)	0.0399 (3)	
N1	0.0604 (2)	0.81235 (19)	0.33473 (18)	0.0687 (5)	
N2	−0.21601 (17)	0.58581 (15)	0.60912 (13)	0.0412 (3)	

### Atomic displacement parameters (Å^2^)

**Table:**

	*U*^11^	*U*^22^	*U*^33^	*U*^12^	*U*^13^	*U*^23^
C1	0.0504 (10)	0.1120 (15)	0.0524 (10)	0.0354 (10)	0.0149 (8)	0.0092 (10)
C2	0.0622 (11)	0.0768 (11)	0.0636 (11)	0.0404 (9)	0.0068 (9)	0.0018 (9)
C3	0.0541 (9)	0.0575 (9)	0.0552 (9)	0.0230 (8)	0.0095 (7)	0.0101 (7)
C4	0.0553 (10)	0.0953 (13)	0.0575 (10)	0.0227 (10)	0.0167 (8)	0.0273 (10)
C5	0.0481 (9)	0.0645 (9)	0.0553 (9)	0.0207 (8)	0.0091 (7)	0.0168 (8)
C6	0.0369 (7)	0.0502 (8)	0.0406 (8)	0.0138 (6)	0.0035 (6)	0.0026 (6)
C7	0.0439 (8)	0.0442 (8)	0.0489 (8)	0.0131 (7)	0.0105 (7)	0.0115 (7)
C8	0.0472 (8)	0.0461 (8)	0.0510 (8)	0.0166 (6)	0.0142 (7)	0.0128 (7)
C9	0.0363 (7)	0.0415 (7)	0.0415 (7)	0.0085 (6)	0.0073 (6)	0.0101 (6)
N1	0.0862 (11)	0.0602 (8)	0.0735 (10)	0.0251 (8)	0.0335 (8)	0.0327 (8)
N2	0.0375 (6)	0.0438 (7)	0.0440 (7)	0.0133 (5)	0.0101 (5)	0.0102 (5)

### Geometric parameters (Å, º)

**Table:**

C1—C2	1.358 (2)	C5—C6	1.381 (2)
C1—C4	1.375 (2)	C5—H5	0.9300
C1—H1	0.9300	C6—C7	1.4551 (19)
C2—C3	1.377 (2)	C7—N2	1.2751 (17)
C2—H2	0.9300	C7—H7	0.9300
C3—C6	1.392 (2)	C8—N1	1.1340 (17)
C3—H3	0.9300	C8—C9	1.4456 (19)
C4—C5	1.375 (2)	C9—C9^i^	1.352 (2)
C4—H4	0.9300	C9—N2	1.3918 (17)
			
C2—C1—C4	120.52 (15)	C4—C5—H5	119.9
C2—C1—H1	119.7	C6—C5—H5	119.9
C4—C1—H1	119.7	C5—C6—C3	118.88 (13)
C1—C2—C3	120.11 (15)	C5—C6—C7	122.30 (13)
C1—C2—H2	119.9	C3—C6—C7	118.82 (14)
C3—C2—H2	119.9	N2—C7—C6	122.49 (13)
C2—C3—C6	120.20 (16)	N2—C7—H7	118.8
C2—C3—H3	119.9	C6—C7—H7	118.8
C6—C3—H3	119.9	N1—C8—C9	175.27 (15)
C1—C4—C5	120.00 (17)	C9^i^—C9—N2	120.38 (15)
C1—C4—H4	120.0	C9^i^—C9—C8	118.58 (14)
C5—C4—H4	120.0	N2—C9—C8	121.04 (11)
C4—C5—C6	120.29 (15)	C7—N2—C9	119.86 (12)
			
C4—C1—C2—C3	−0.5 (3)	C2—C3—C6—C7	179.53 (14)
C1—C2—C3—C6	0.5 (3)	C5—C6—C7—N2	−0.7 (2)
C2—C1—C4—C5	−0.1 (3)	C3—C6—C7—N2	179.89 (12)
C1—C4—C5—C6	0.7 (3)	C6—C7—N2—C9	−179.29 (12)
C4—C5—C6—C3	−0.6 (2)	C9^i^—C9—N2—C7	178.32 (15)
C4—C5—C6—C7	179.93 (14)	C8—C9—N2—C7	−1.5 (2)
C2—C3—C6—C5	0.1 (2)		

Symmetry code: (i) −*x*, −*y*+1, −*z*+1.
